# Supplementary figures and images for: The molecular determinants of a universal prion acceptor
Source: PLoS Pathog. 2024 Sep 10;20(9):e1012538. doi: 10.1371/journal.ppat.1012538 (PMC11414987; doi:10.1371/journal.ppat.1012538)

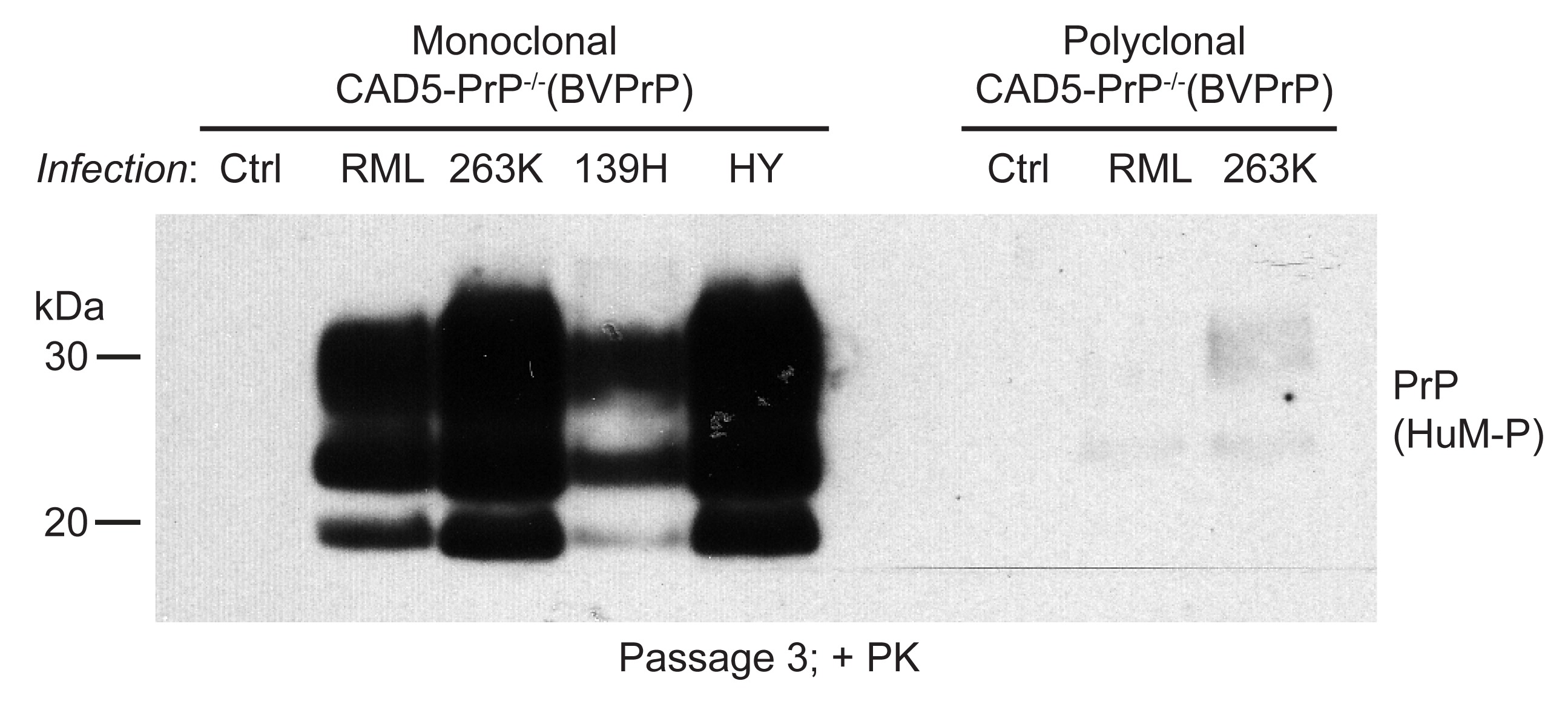

Supplement: S1 Fig — Immunoblot of PrPres levels in monoclonal and polyclonal lines of stably transfected CAD5-PrP-/-(BVPrP) cells infected with either mouse (RML) or hamster (263K, 139H, or HY) prion strains. Cells were analyzed at passage 3 post-infection. PrPres was detected using the antibody HuM-P. Molecular weight markers indicate kDa. (TIF) [file ppat.1012538.s001.tif]

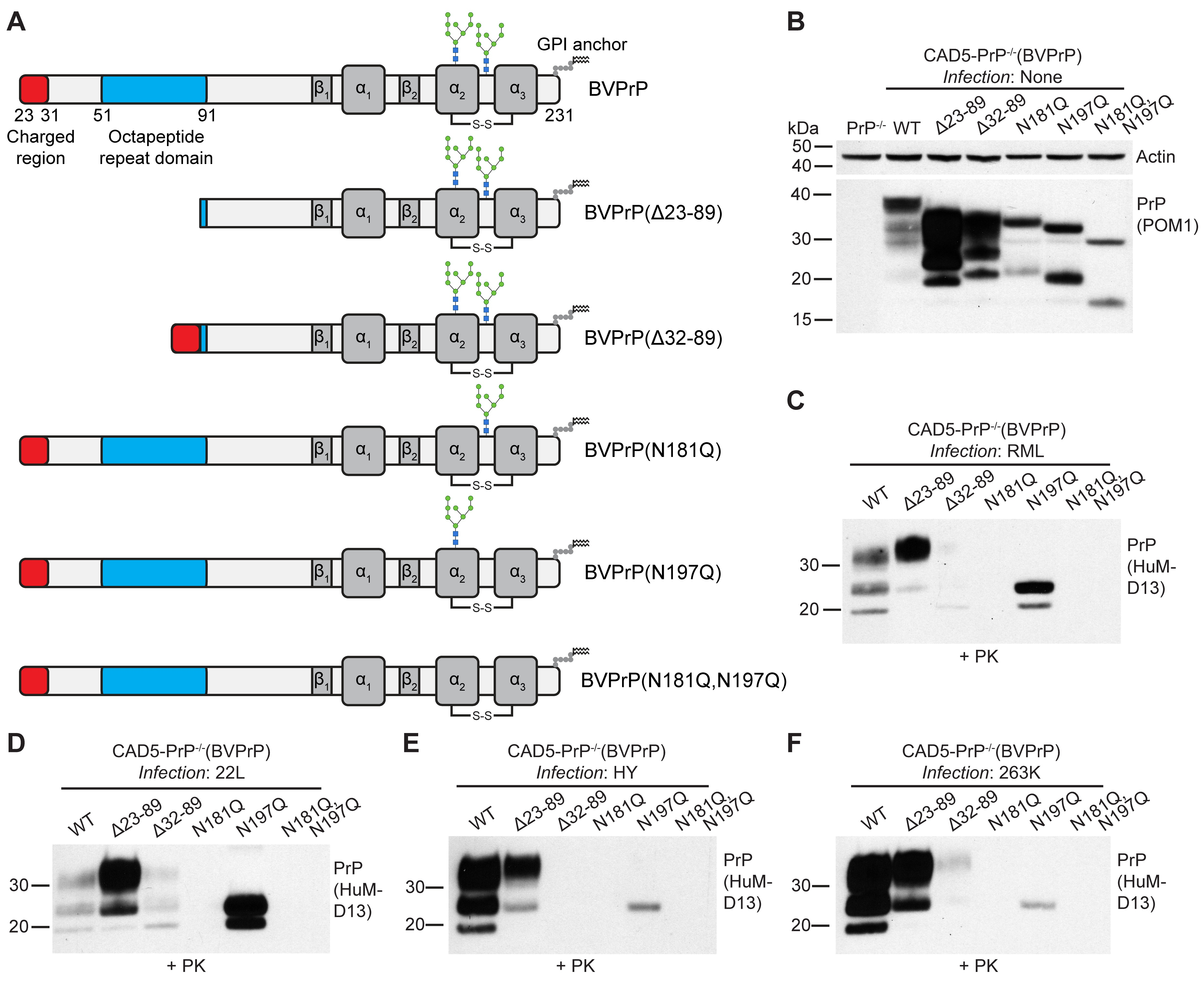

Supplement: S2 Fig — (A) Domain structures of the BVPrP constructs used. For simplicity, the N- and C-terminal signal sequences are not depicted in the diagram. All constructs contain methionine at codon 109. (B) Immunoblot for PrPC in undigested lysates from CAD5-PrP-/- cells stably expressing the indicated BVPrP constructs. The blot was reprobed with an antibody against actin. (C-F) Immunoblots of PrPres levels in CAD5-PrP-/- cells stably expressing the indicated BVPrP constructs challenged with either mouse RML (C), mouse 22L (D), hamster HY (E), or hamster 263K (F) prions. PrPC was detected using the antibody POM1 whereas PrPres was detected using HuM-D13. In all panels, the molecular weight markers indicate kDa. (TIF) [file ppat.1012538.s002.tif]

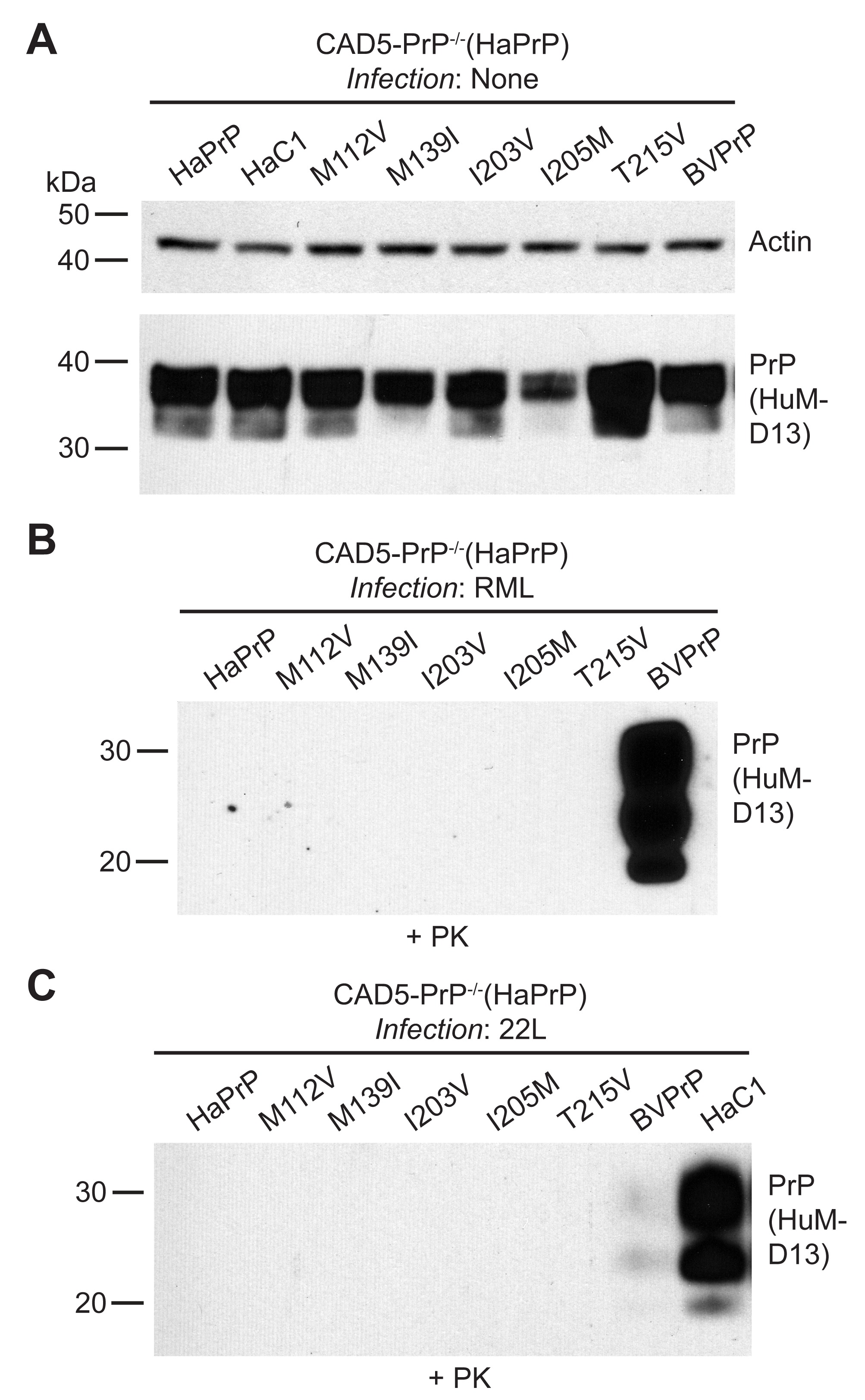

Supplement: S3 Fig — (A) Immunoblot for PrPC in undigested lysates from CAD5-PrP-/- cells stably expressing wild-type HaPrP or BVPrP, the HaC1 chimera, or HaPrP with the indicated BVPrP residue substitutions. The blot was reprobed with an antibody against actin. (B-C) Immunoblots of PrPres levels in lysates from CAD5-PrP-/- cells stably expressing the indicated PrPs challenged with either RML (B) or 22L (C) mouse prions. PrP was detected using the antibody HuM-D13. In all panels, the molecular weight markers indicate kDa. (TIF) [file ppat.1012538.s003.tif]

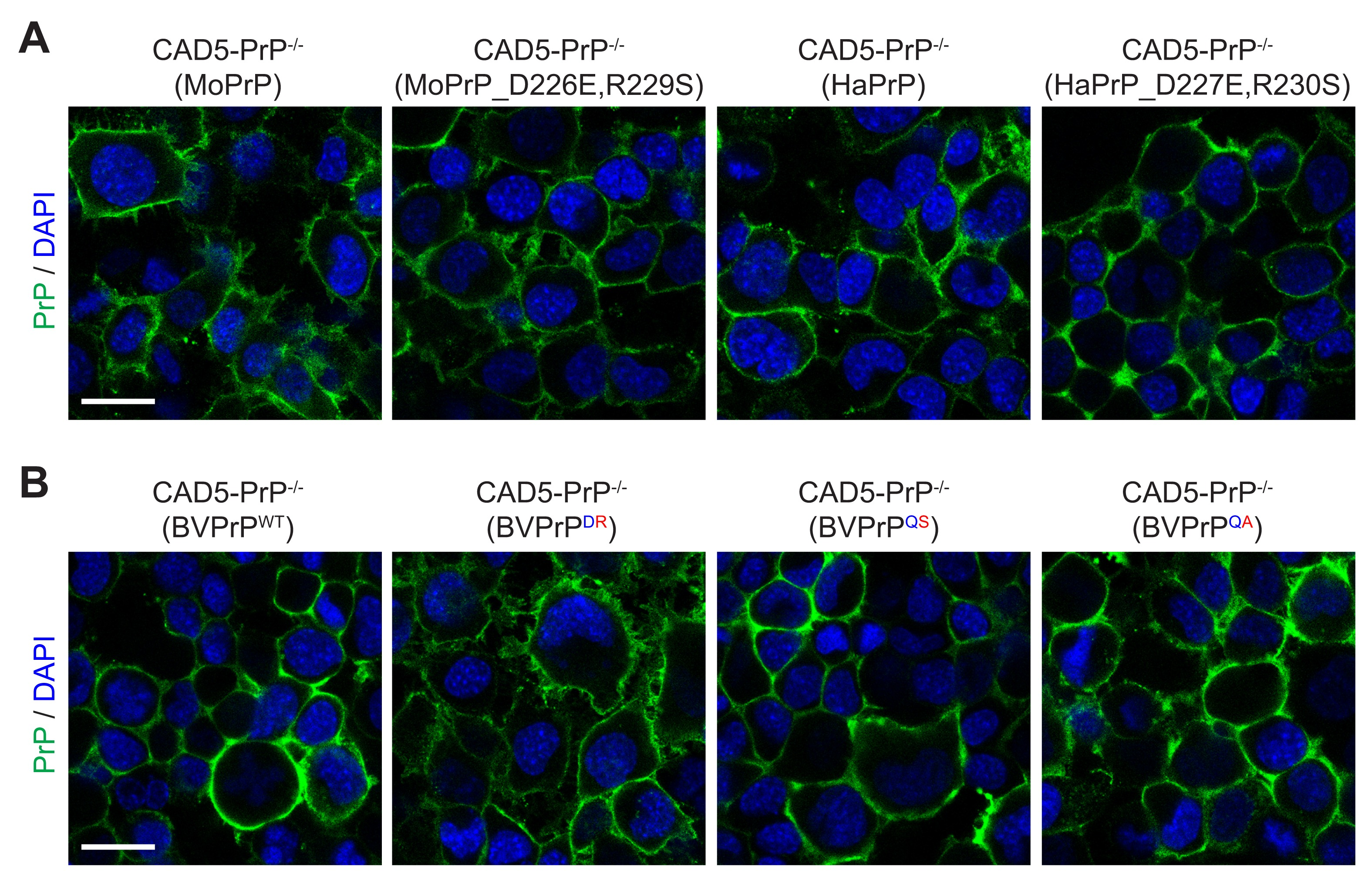

Supplement: S4 Fig — (A) Immunofluorescence images of non-permeabilized stably transfected polyclonal CAD5-PrP-/- lines expressing either wild-type MoPrP, wild-type HaPrP, or mutants of MoPrP and HaPrP in which the indicated C-terminal residues were substituted for their BVPrP equivalents. (B) Immunofluorescence images of non-permeabilized stably transfected polyclonal CAD5-PrP-/- lines expressing either wild-type BVPrP or the indicated BVPrP C-terminal chimeras. In both panels, cell surface PrP expression (green) was revealed using the antibody POM1 and nuclei were stained with DAPI (blue). Scale bars = 25 μm. (TIF) [file ppat.1012538.s004.tif]

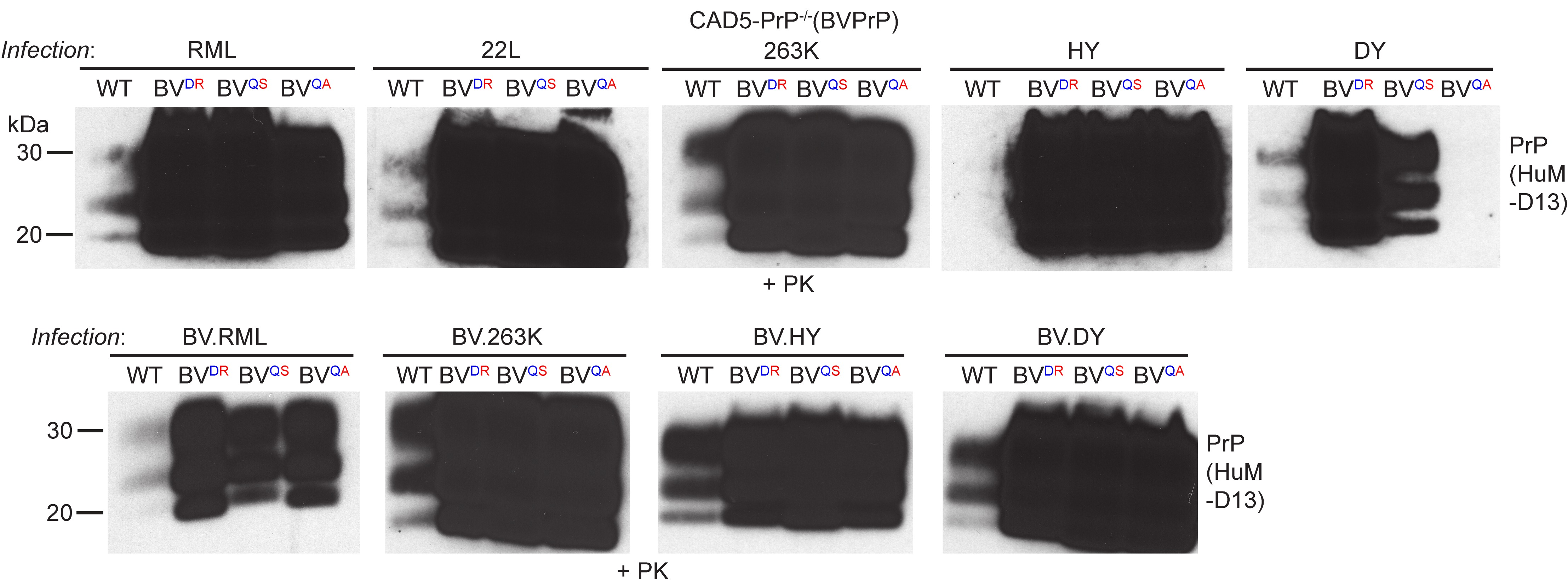

Supplement: S5 Fig — Immunoblots of PrPres levels in lysates from CAD5-PrP-/- cells stably expressing the indicated PrP molecules challenged with either mouse, hamster, or bank vole-adapted prion strains. PrPres was detected using the antibody HuM-D13. The molecular weight markers indicate kDa. (TIF) [file ppat.1012538.s005.tif]

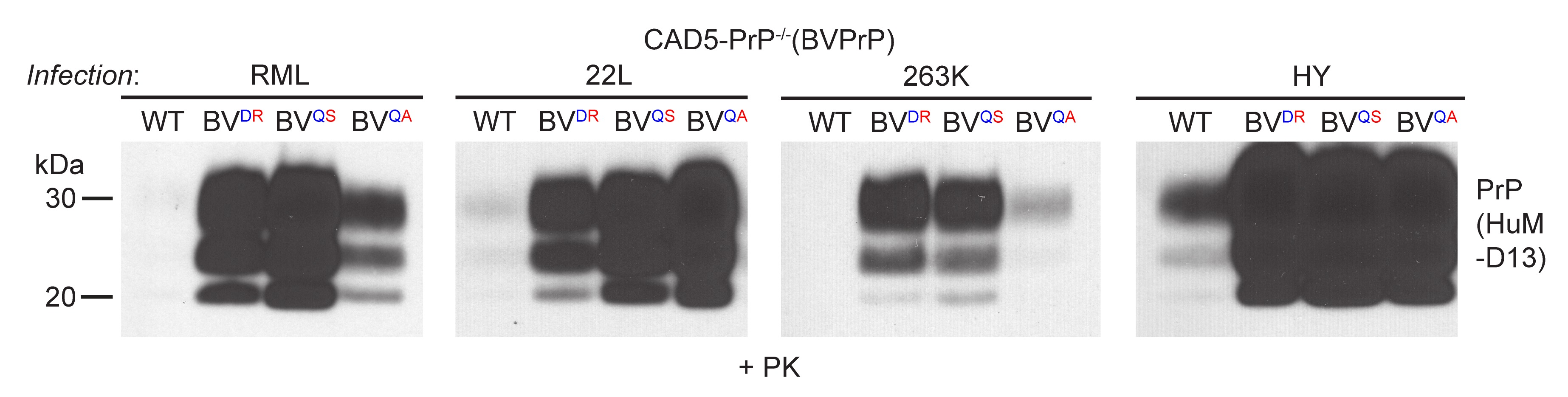

Supplement: S6 Fig — Immunoblots of PrPres levels in lysates from CAD5-PrP-/- cells stably expressing the indicated PrP molecules challenged with either mouse or hamster prion strains. PrPres was detected using the antibody HuM-D13. The molecular weight markers indicate kDa. (TIF) [file ppat.1012538.s006.tif]
